# Diagnostic accuracy of methylated *SEPT*9 for primary liver cancer: a systematic review and meta-analysis

**DOI:** 10.3389/fendo.2025.1434174

**Published:** 2025-02-13

**Authors:** Danwen Jin, Liyong Qian, Jiayao Chen, Ze Yu, Jinliang Dong

**Affiliations:** ^1^ Pathological Diagnosis Center, Zhoushan Hospital, Zhoushan, Zhejiang, China; ^2^ Department of Laboratory, Zhoushan Hospital, Zhoushan, Zhejiang, China; ^3^ Laboratory of Cell Biology and Molecular Biology, Zhoushan Hospital, Zhoushan, Zhejiang, China; ^4^ Department of Hepatobiliary Surgery, Zhoushan Hospital, Zhoushan, Zhejiang, China

**Keywords:** diagnostic performance, methylation, SEPT9, primary liver cancer, meta-analysis

## Abstract

**Background:**

Primary live cancer (PLC), including hepatocellular carcinoma (HCC) and intrahepatic cholangiocarcinoma (ICC). This meta-analysis was conducted to evaluate the diagnostic efficacy of blood methylated septin 9 gene (*mSEPT9*) for PLC and to analyze its performance across various subgroups.

**Methods:**

We conducted a comprehensive search across PubMed, the Cochrane Library, Embase, and China National Knowledge Infrastructure (CNKI), covering research up to May 10, 2024. The pooled sensitivity, specificity, diagnostic odds ratios, and area under the summary receiver operating characteristic (AUC) were calculated for the diagnostic performance of *mSEPT9* for PLC. The quality of the studies was assessed using the QUADAS-2 tool, and the meta-analysis was performed using Stata16.0 software.

**Results:**

Ten articles with 2,182 participants were included in the meta-analysis. The pooled sensitivity of *mSEPT9* for detecting primary liver cancer was 0.51 (95% confidence interval [CI]: 0.37-0.65), and the pooled specificity was 0.93 (95% CI: 0.78-0.98). The pooled diagnostic odds ratio was 13 (95% CI: -58), and the area under the Summary Receiver Operator Characteristic Curve was 0.75 (95% CI: 0.71-0.79). Subgroup analyses showed that ICC, case-control studies, qPCR and Asian populations had higher specificities (0.99 [95% CI: 0.97–1.00], 0.93 [95% CI: 0.91-0.95], 0.90 [95% CI: 0.88-0.92] and 0.94 [95% CI: 0.92-0.96], respectively) and diagnostic odds ratios (62.04 [95% CI: 6.53-589.53], 17.62 [95% CI: 4.03-76.99], 13.03 [95% CI: 2.01-84.63] and 14.19 [95% CI: 2.42-83.11], respectively) compared to hepatocellular carcinoma, cohort Study, and Euramerican populations.

**Conclusions:**

This study confirmed that *mSEPT9* in blood has high specificity and moderate sensitivity for detecting primary liver cancer. The diagnostic performance of *mSEPT9* varied across different subgroups, limiting its use as an independent screening tool and necessitating its use in conjunction with other methods for confirmatory diagnostics.

**Systematic review registration:**

https://www.crd.york.ac.uk/prospero/, identifier CRD42024549669.

## Introduction

Primary liver cancer (PLC), including hepatocellular carcinoma (HCC) and intrahepatic cholangiocarcinoma (ICC), represents a major public health challenge globally given that it is the sixth most prevalent cancer and the third cause of cancer-related deaths across the globe. It kills around 830K people a year (1). The geographic variability in the incidence of liver cancer is explained in part by the distribution of risk factors, such as chronic infection with hepatitis B or C viruses, aflatoxin exposure, alcohol drinking, and nonalcoholic fatty liver disease. Greater rates of incidence are observed in Eastern Asia and sub-Saharan Africa, due to the high prevalence of chronic hepatitis B infection in those regions. Liver cancer has a dismal prognosis despite advances in early diagnosis and treatment, with a five-year survival rate of <20% in most regions, highlighting the need for innovative diagnostic and therapeutic strategies (2).

PLC is usually diagnosed using imaging techniques (such as ultrasound, computed tomography (CT), or magnetic resonance imaging (MRI) scans and tests for levels of substances (such as alpha-fetoprotein, AFP) in the plasma and/or serum (3). Recently, the era of liquid biopsy opened new possibilities for the non-invasive diagnosis of liver cancer, with expected improvements in sensitivity and specificity. Liquid biopsies, however, provide a dynamic snapshot of the genetics of the tumor and the tumor microenvironment by analyzing circulating tumor cells (CTCs), cell-free DNA (cfDNA), and other biomarkers from a blood sample (4, 5). It has shown promise, particularly in early detection and response and progression monitoring of treatment, highlighted by its enhanced ability to detect molecular sub-steps of tumor genesis and heterogeneity compared to conventional strategies. Recent studies also show that liquid biopsies aid in an appropriate and accurate diagnosis, reducing unnecessary imaging methods and supporting the clinical utility of liquid biopsies for liver cancer (6, 7).

Methylated septin 9 gene (*mSEPT9*), initially known for its promise in non-invasively indicating the presence of colorectal cancer, has also been investigated for its relevance in multiple other cancers including liver cancer (8). The pattern of methylation adducts in the gene results in its abnormal silencing and is a compelling tumor suppressor gene critical in cellular cytoskeleton organization as well as cell division (9–11). Its use for diagnosing liver cancer shows promise, in part because in initial studies it has shown a high level of sensitivity and specificity. *SEPT9* protein expression examined by immunohistochemical (IHC) was significantly different among various forms of hepatic nodules. We demonstrated that *SEPT9* expression was significantly correlated with advanced tumor grade and concurrent *SATB2* staining in HCC tissues, which supported the importance of *SEPT9* in liver carcinogenesis (12). This may highlight how the septic protein is specific in cancer because there was no *SEPT9* expression in dysplastic nodes, occasionally in hepatocellular adenoma, in contrast to the non-expression in benign hepatocytes (13). In addition, this approach allows early detection of disease states and may be used to track disease (re)occurrence and response to therapy, making it an ideal candidate for current and future diagnostic strategies (14, 15). However, despite the promising potential of *mSEPT9* in liver cancer diagnosis, it should be noted that the clinical application of *mSEPT9* testing in most malignancies has not been established. Even in colorectal cancer, where *mSEPT*9 testing has been used clinically, significant drawbacks were observed, including high operational costs and unsatisfactory sensitivity (16).

The evolving landscape of liver cancer research increasingly supports the use of molecular biomarkers that offer both specificity and non-invasiveness as emerging diagnostic solutions. The methylation of the *SEPT9* gene is the most promising target of these markers since it has already shown important diagnostic performance for colorectal cancer and seems to be of potential relevance also for liver cancer. We conducted an exploratory meta-analysis to summarize the diagnostic accuracy of *mSEPT9* in patients suspected of primary liver cancer and to analyze its performance across various subgroups, with the aim of identifying areas for further research and refinement.

## Methods

### Study design

This systematic review was conducted and reported according to the Preferred Reporting Items for Systematic Reviews and Meta-Analyses (PRISMA) checklist (17). This study was based on a secondary analysis of published literature and therefore does not require ethical review approval. The study has been registered on the international prospective register of systematic reviews under registration number CRD42024549669.

### Information sources and search strategy

Two authors (DJ and ZY) independently performed the abstract screen and full-text review, with disagreements resolved by consensus. Literature search was carried out in electronic databases as follows: PubMed, Cochrane Library, Embase, and China National Knowledge Infrastructure (CNKI). The search strategy was aimed at retrieving the most relevant items published up to the search date of this review and these articles were only included if they were published in English and Chinese. Aforementioned, the databases were searched electronically, and reference lists of relevant articles and review papers were also hand-searched to reduce the chances of missing out on the identifiable potential studies during the electronic search. We established a comprehensive search strategy to search for the studies regarding the diagnostic accuracy of *mSEPT9* for primary liver cancer. In particular, the following keywords and search strings were employed: “methylated SEPT9”, “SEPT9 methylation”, “SEPT9”, “Septin9”, “Septin 9”, “serum”, “plasma”, “blood”, “liver cancer”, “liver tumour”, “liver carcinoma”, “liver neoplasms”, “hepatocellular tumour”, “hepatocellular carcinoma”, “hepatoma”. These terms were then combined using Boolean operators (AND, OR). Search strings used for each database are provided in **Supplementary Table S1**.

### Inclusion and exclusion criteria

To be eligible for inclusion in the systematic review and meta-analysis, studies had to meet the following criteria: 1) evaluate the diagnostic performance of *mSEPT9* for primary liver cancer (PLC) in adult human populations; 2) provide sufficient data to estimate sensitivity, specificity, and other diagnostic accuracy parameters, including true positive, false positive, true negative, and false negative values; 3) adopt histopathological or imaging as standard reference standard for diagnosis; 4) be accessible in full text, and 5) be published in a scientific journal. Exclusion criteria included: 1) reviews, case reports, letters, conference abstracts, and non-human studies; 2) articles that did not present original research data (e.g., editorials, commentaries); 3) Studies with unclear or inconsistent cut-off thresholds for *mSEPT9*, as variations in cut-off points could significantly impact diagnostic performance and limit comparability; 4) studies with populations that did not match the intended patient profile (e.g., non-PLC conditions, pediatric populations, or patients with other advanced liver diseases).

### Data extraction

Data extraction was conducted by two authors (LQ and JC), independently, based on a predefined excel sheet, including the first author name, publication year, country, study period, study design, total number of patients, patient population characteristics (with reference to specific pathology such as HCC or ICC), mean age, gender distribution, diagnostic methods, methods for detecting *SEPT9* methylation, test cut-off values, and the number of *SEPT9* positive cases. We also extracted the true positives (TP), false positives (FP), false negatives (FN), and true negatives (TN) to calculate sensitivity, specificity, positive and negative likelihood ratios (PLR and NLR), and diagnostic scores.

### Quality assessment

Two reviewers (LQ and JC) independently assessed quality and resolved disagreements by discussion or by consulting a third reviewer. All studies included in this systematic review and meta-analysis were evaluated by the Quality Assessment of Diagnostic Accuracy Studies-2 (QUADAS-2) tool. QUADAS-2 is a major methodological tool of accepted standard specifically for risk of bias and applicability concerns in diagnostic accuracy studies. The evaluation was divided by four principle fields: patient selection, index test, reference standard, and flow and timing. Risk of bias was assessed for each domain, and the first three domains were also evaluated for concerns regarding applicability to the research question.

### Statistical analysis

The identification accuracy of liver cancers was evaluated via the statistical software Stata 16.0 (Stata Corp LLC) in this study. The summary receiver operating characteristic (SROC) curve provided a global measure of test effectiveness. For each study, the diagnostic odds ratio (DOR) was calculated. Heterogeneity was assessed by the I2 statistic, with an I2 value of less than 25% indicating mild heterogeneity, I^2^ value from 25% to 50% indicating moderate heterogeneity, and an I^2^ value of more than 50% indicating significant heterogeneity. A fixed-effects model using the Mantel-Haenszel method was applied in cases of mild heterogeneity, while a random-effects model utilizing the DerSimonian and Laird method was employed for greater heterogeneity. Fagan’s nomogram was utilized to translate likelihood ratios into clinically relevant post-test probabilities. Publication bias was evaluated using Deeks’ funnel plot asymmetry test. Additionally, a distribution scatter diagram visualized the spread of diagnostic accuracies across studies, and subgroup analyses explored the sources of heterogeneity based on study characteristics such as pathology, design of the study, test method and nationality. To further ensure the robustness of our findings, we conducted a sensitivity analysis, systematically excluding each study to observe changes in the overall effect estimate.

## Results

### Study selection

**Figure 1:**
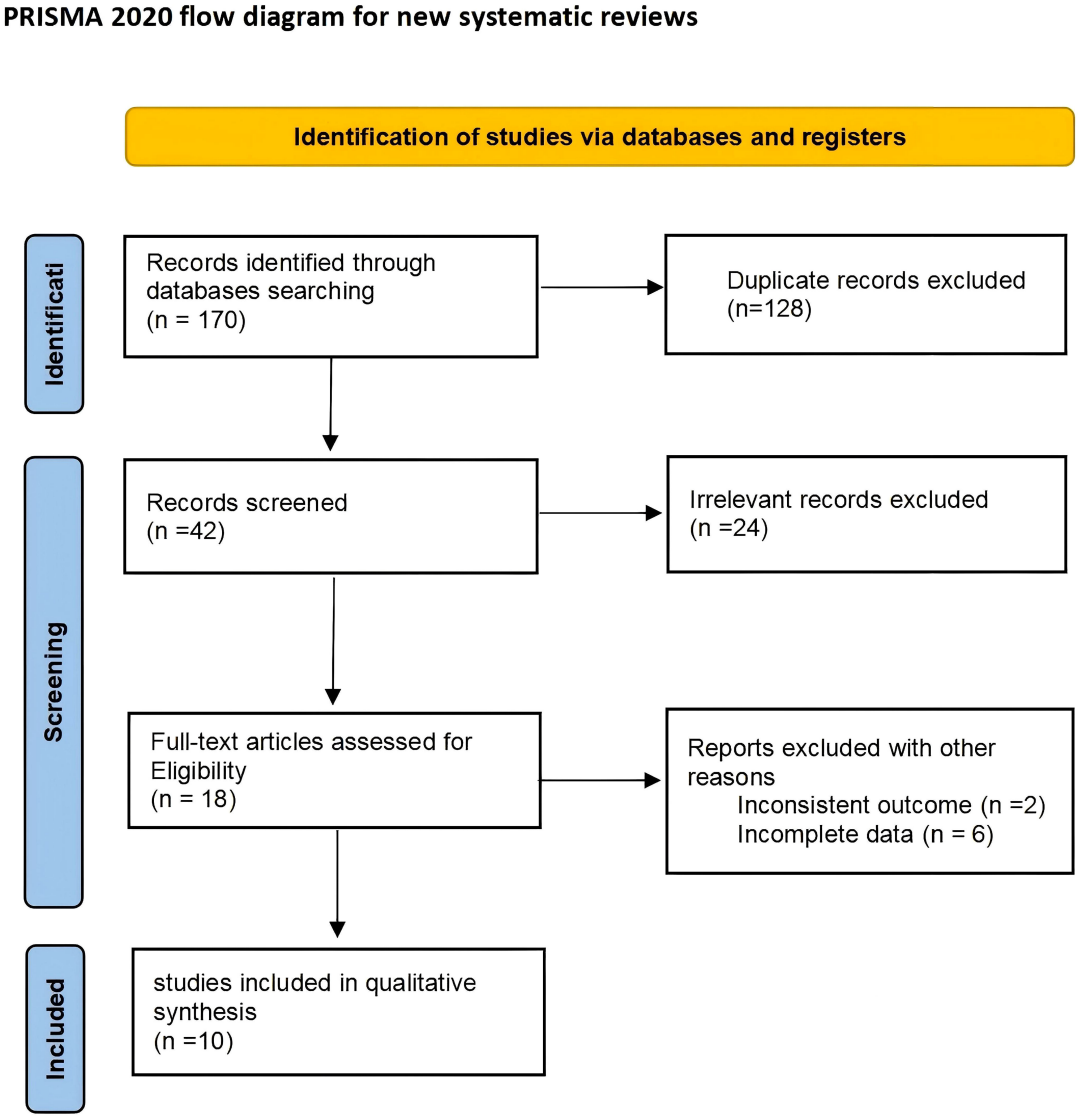
Flow diagram of study selection.

A total of 170 articles were identified in the initial search. 128 of these were excluded due to duplication or non-adherence to the inclusion criteria. Another search in the titles and abstracts provided 24 of the articles to be excluded. The full text of remaining 18 articles were further reviewed in which eight articles were excluded from the review as two studies failed to detail their diagnostic test and six did not have enough data to calculate the sensitivity and specificity. In total, 10 studies met the inclusion criteria for this meta-analysis (**Figure 1**).

### Characteristics of included studies

This systematic review covers 10 studies from 2012 to 2023 consisting of 2,182 participants with the majority derived from HCC and a smaller portion from ICC (13, 18–26). The dataset used in this study covered various countries, including France, Germany, the United Kingdom (UK), China, Japan, and the United States of America (USA). Among the included studies, 7 were case-control studies, 2 was a cohort study, and the remaining one study consisted of a cohort study (initial) and a case-control study (replication). The mean age of participants was from 53.9 to 68.3 years and gender distribution was also different between studies, most having a higher percentage of men. Main diagnostic methods of PLC include biopsy, CT, MRI, and ultrasound examination. The volume of blood samples collected from patients varies from 0.4 ml to 10 ml. In the detection of *mSEPT9*, seven studies employed quantitative polymerase chain reaction (qPCR), while the remaining three studies utilized multiplex droplet digital PCR (ddPCR), multiplex PCR, and IHC, respectively. There was wide variability in the cut-off values to define positive *SEPT9* methylation cases, ranging from discernable cycle threshold values to the quantification of gene copies. The number and percentage of positive cases in each study varied from 38 to 276 and from 17.7% to 62.9%, which were demonstrated in **Table 1**.

**Table 1:** Characteristics of included studies.

Author	Country	Study period	Study design	No. of patients	Pathological type	Age	Gender (M/F)	Diagnosis method	Blood volume	Test method	Cut-off value	Positive cases	TP	FP	FN	TN
Oussalah, 2018 (21)	France and German	2012-2016	Cohort Study/Case–control	289	HCC	Mean (sd), 62.3 ± 10.9	219/70	Biopsy + CECT	10 ml	qPCR	More than 2 triplicates	98, 33.9%	89	27	164	9
Bannaga, 2020 (22)	UK	2013-2019	Cohort Study	141	HCC	Mean, 60.7	81/60	Biopsy	1 ml	qPCR	Cycle threshold value of 32.1	38, 27.0%	34	20	83	4
Shen, 2020 (23)	China	2018-2019	Case–control	439	HCC, ICC	Mean (sd), 58.7 ± 8.8	310/129	Biopsy	NR	qPCR	Cycle threshold value of 32.1	276, 62.9%	148	0	128	163
He, 2020 (24)	China	2016-2017	Case–control	362	HCC	Mean, 53.9	270/92	Biopsy + CT	10 ml	qPCR	ACTB threshold count of 41.1	64, 17.7%	49	12	286	15
Kotoh, 2020 (25)	Japan	2015-2018	Case–control	216	HCC	Mean (sd), 64.1 ± 8.5	136/80	Biopsy + imaging	0.4 ml	Multiplex ddPCR	4.6 copies	136, 63.0%	86	8	72	50
Li, 2020 (26)	China	2018-2019	Case-control	278	HCC	>50, 162, 58.3%; ≤50, 116, 41.7%	183/95	Biopsy + CT, MRI, ultrasonic	10 ml	qPCR	CT cut-off value of 41	104, 37.4%	86	7	167	18
Lewin, 2021 (27)	Germany	NR	Case–control	163	HCC	Mean (sd), 58.9 ± 10.2	87/76	Biopsy + CT/MRI	3.5 ml	Multiplex PCR	NR	60, 36.8%	46	14	66	37
Liu, 2023 (28)	China	2021-2022	Case–control	100	HCC, ICC	Mean (sd), 68.3 ± 3.5	53/47	Biopsy	10 ml	qPCR	Cycle threshold value of 41	60, 60.0%	32	1	28	39
Kmeid, 2023 (15)	USA	2003-2021	Case–control	122	HCC	Mean, 56.3	68/55	Biopsy + imaging	NR	IHC	Membranous accentuation in 5% of tumor cells	68, 55.7%	22	1	46	53
Zheng, 2023 (29)	China	NR	Case–control	172	HCC	> 50, 129, 75.0%; ≥ 50, 43, 25.0%	130/42	Biopsy + CECT/MRI	10 ml	qPCR	cycle threshold value of -2.7	51, 29.7%	33	9	18	112

HCC, hepatocellular carcinoma; ICC, intrahepatic cholangiocarcinoma; CECT, contrast-enhanced computed tomography; qPCR, quantitative real-time polymerase chain reaction; ddPCR, droplet digital polymerase chain reaction; UK, the United Kingdom; NR, no reported; IHC, immunohistochemical.

### Quality assessment

**Supplementary Figure S1** displays the quality assessment results for all included studies. In the assessment of risk of bias, most studies exhibited low risk. However, some studies were unclear in the areas of “patient selection” (n=5), “index testing” (n=4), and “flow and time” (n=3). Meanwhile, applicability concerns were present for most studies toward the patient selection (n=8), index test (n=4), but not reference standard (n=8).

### Overall diagnostic value of *mSEPT9* for primary liver cancer

**Figure 2:**
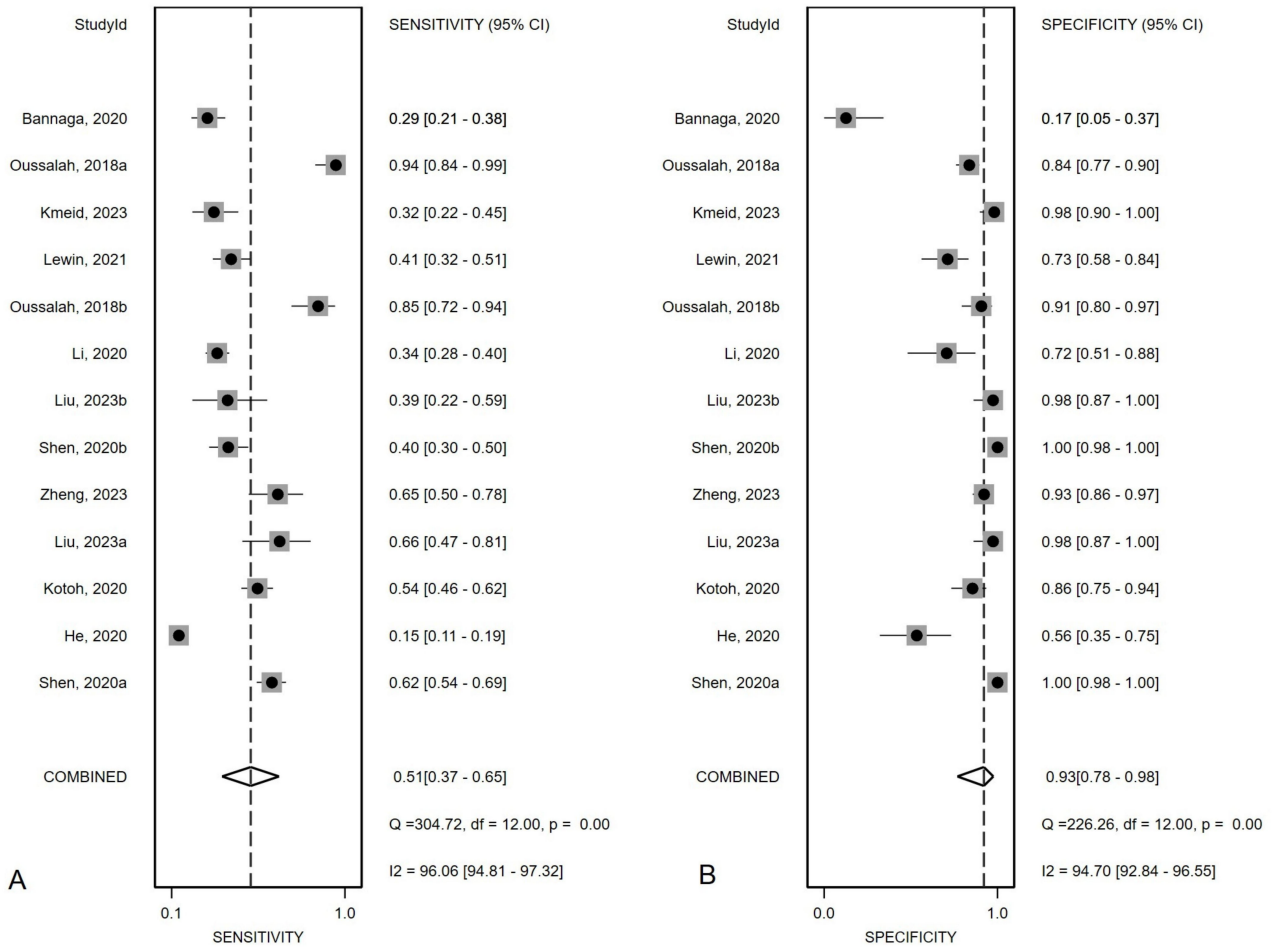
Summary sensitivity **(A)** and specificity **(B)** plotted on forest graphs for *mSEPT9* in diagnosis for primary liver cancer.

**Figure 3:**
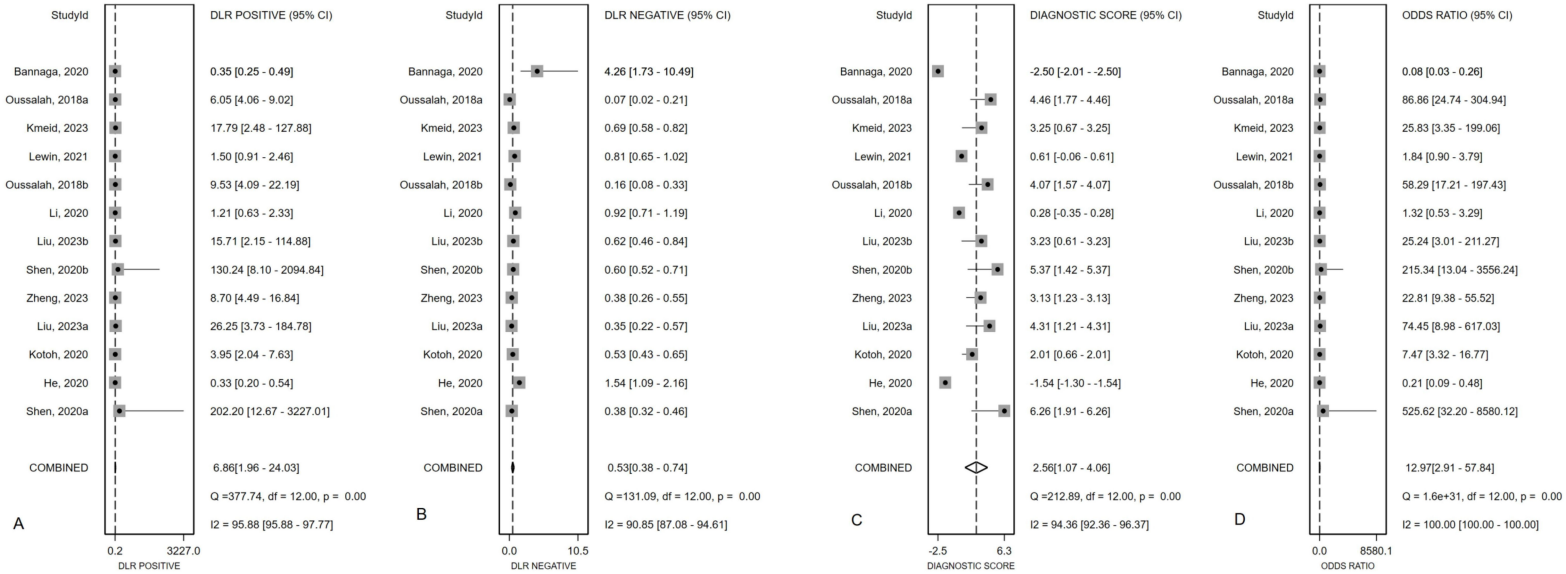
Summary PLR **(A)**, NLR **(B)**, diagnostic score **(C)** and DOR **(D)** plotted on forest graphs for *mSEPT9* in diagnosis for primary liver cancer.

**Figure 4:**
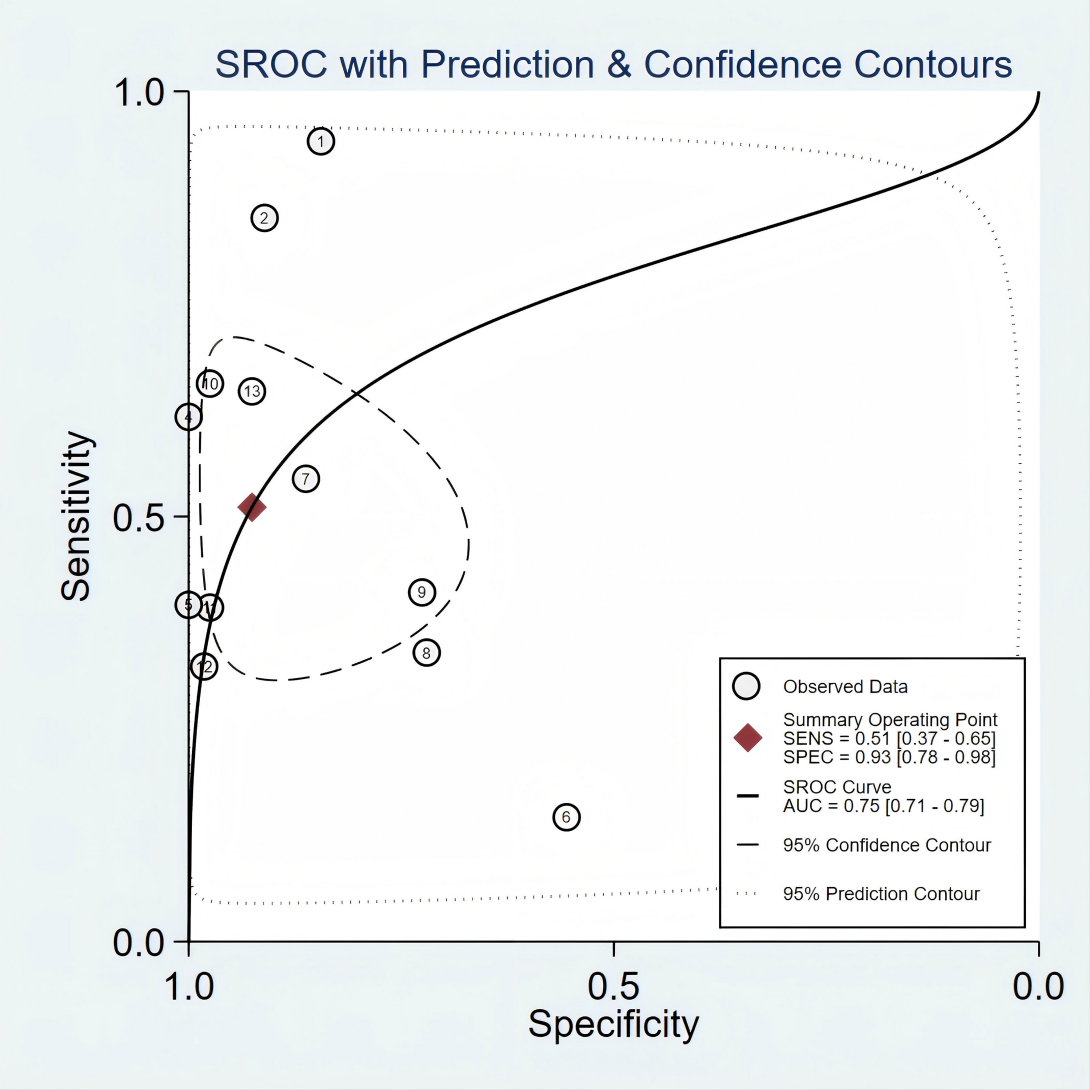
SROC curve for *mSEPT9* in diagnosis of primary liver cancer.

**Figure 2** presented the forest plot depicting the pooled sensitivity and specificity of *mSEPT9* in diagnosing primary liver cancer, which were 0.51 (95% confidence interval (CI): 0.37–0.65) and 0.93 (95% CI: 0.78–0.98), respectively. Significant heterogeneity was observed among the included studies, with I² values of 96.06 (95% CI: 94.81–97.32) for sensitivity and 94.70 (95% CI: 92.84–96.55) for specificity. Additionally, PLR, NLR, diagnostic score, and DOR were reported as 6.86 (95% CI: 1.96–24.03), 0.53 (95% CI: 0.38–0.74), 2.56 (95% CI: 1.07–4.06), and 12.97 (95% CI: 2.91–57.84), respectively, as illustrated in **Figure 3**. The area under the curve (AUC) for *mSEPT9* and primary liver cancer was 0.75 (95% CI: 0.71–0.79), shown in **Figure 4**. The Spearman correlation coefficient of -0.311 (p = 0.301) indicated that there was no threshold effect in the analysis.

### Subgroup analyses

Subgroup analyses of diagnostic test performance metrics by pathological type, study design, geographic area, and test method (**Table 2**). For pathological types, *mSEPT9* testing demonstrated distinct diagnostic performances in HCC and ICC. In HCC patients, *mSEPT9* showed a sensitivity of 0.41 (95% CI: 0.38-0.44) and a specificity of 0.87 (95% CI: 0.84-0.89), with a DOR of 7.89. This performance is characterized by high heterogeneity (I² = 96.6%). On the other hand, ICC displayed a slightly lower sensitivity at 0.40 (95% CI: 0.31-0.49), but a notably higher specificity of 0.99 (95% CI: 0.97-1.00). The DOR for ICC was exceptionally high at 62.04, accompanied by substantially lower heterogeneity. Regarding study design, case-control studies showed a sensitivity of 0.41 (95% CI: 0.38-0.44) and a higher specificity of 0.93 (95% CI: 0.91-0.95), with a DOR of 17.62. Cohort studies displayed a lower sensitivity of 0.40 (95% CI: 0.35-0.45) and specificity of 0.74 (95% CI: 0.67-0.80), with a DOR of 2.09. Geographically, studies conducted in Asia demonstrated a sensitivity of 0.38 (95% CI: 0.36-0.41) and higher specificity of 0.94 (95% CI: 0.92-0.96), resulting in a DOR of 14.19. Conversely, studies from Euramerican regions showed a sensitivity of 0.48 (95% CI: 0.43-0.53) and a lower specificity of 0.81 (95% CI: 0.76-0.85), with a DOR of 6.98. For test method, qPCR has a sensitivity of 0.40 (0.37- 0.42) and a specificity of 90.90 (0.88- 0.92). Other methods reported a sensitivity of 0.46 (0.40- 0.51) and a specificity of 0.86 (0.80- 0.91), showing slightly higher sensitivity but lower specificity than qPCR. The DOR further underscores the effectiveness, with qPCR at 13.03, significantly higher than 5.64 for other methods.

**Table 2:** The results of subgroup analysis.

Analysis	No.	Sensitivity	I^2^, %	Specificity	I^2^, %	PLR	I^2^, %	NLR	I^2^, %	DOR	I^2^, %
Pathological type											
HCC	11	0.41 (0.38-0.44)	96.6%	0.87 (0.84-0.89)	93.5%	3.94 (1.34-11.58)	96.9%	0.57 (0.41-0.79)	92.7%	7.89 (1.89-32.91)	94.7%
ICC	2	0.40 (0.31-0.49)	0.0%	0.99 (0.97-1.00)	69.4%	37.49 (3.90-360.44)	44.8%	0.61 (0.53-0.70)	0.0%	62.04 (6.53-589.53)	40.5%
Study design											
Case–control	10	0.41 (0.38-0.44)	95.8%	0.93 (0.91-0.95)	91.5%	8.71 (2.44-31.17)	94.9%	0.55 (0.43-0.71)	90.0%	17.62 (4.03-76.99)	93.1%
Cohort Study	3	0.40 (0.35-0.45)	97.4%	0.74 (0.67-0.80)	95.4%	1.37 (0.19-10.12)	98.4%	0.67 (0.01- 4.68)	95.2%	2.09 (0.055-78.96)	97.0%
Area											
Euramerica	5	0.48 (0.43-0.53)	96.2%	0.81 (0.76-0.85)	94.3%	3.20 (0.66-15.45)	97.6%	0.52 (0.26- 1.05)	93.8%	6.98 (0.57-85.97)	95.7%
Asia	8	0.38 (0.36-0.41)	96.0%	0.94 (0.92-0.96)	92.4%	8.13 (1.66-39.82)	95.8%	0.59 (0.45- 0.79)	90.4%	14.19 (2.42-83.11)	93.9%

HCC, hepatocellular carcinoma; ICC, intrahepatic cholangiocarcinoma.

### Publication bias

**Figure 5:**
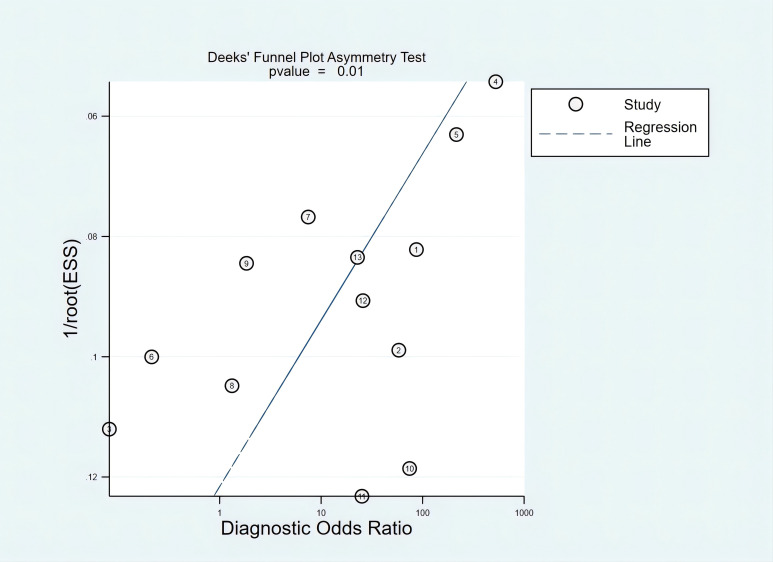
Fagan plots for assessing the clinical utility.

The asymmetry in the plot, as indicated by several studies deviating significantly from the regression line, was statistically significant, with a *p*-value of 0.01. This suggested the presence of publication bias (**Figure 5**).

### Clinical diagnostic value

**Figure 6:**
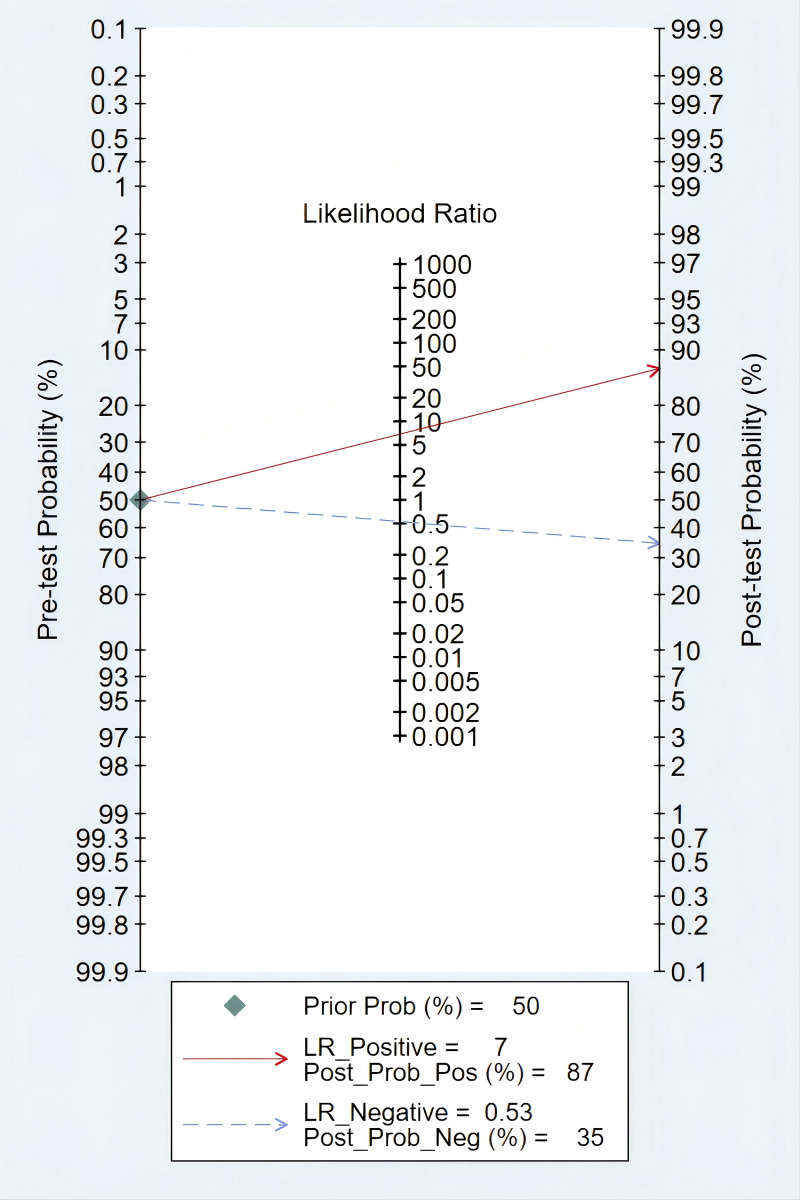
Deek’s funnel plot asymmetry test of *mSEPT9* in diagnosis for primary liver cancer.

According to Fagan’s nomogram, our findings indicated that a positive *mSEPT9* test result increased the post-test probability of an individual having PLC to approximately 87%, assuming a pre-test probability of 50%. This suggested that if a patient tested positive for PLC using *mSEPT9*, the likelihood that they truly had the disease was significantly elevated to 87%. Conversely, a negative test result reduced the post-test probability to around 35%, indicating that a negative *mSEPT9* test could effectively rule out the disease with a high degree of certainty (**Figure 6**).

**Figure 7:**
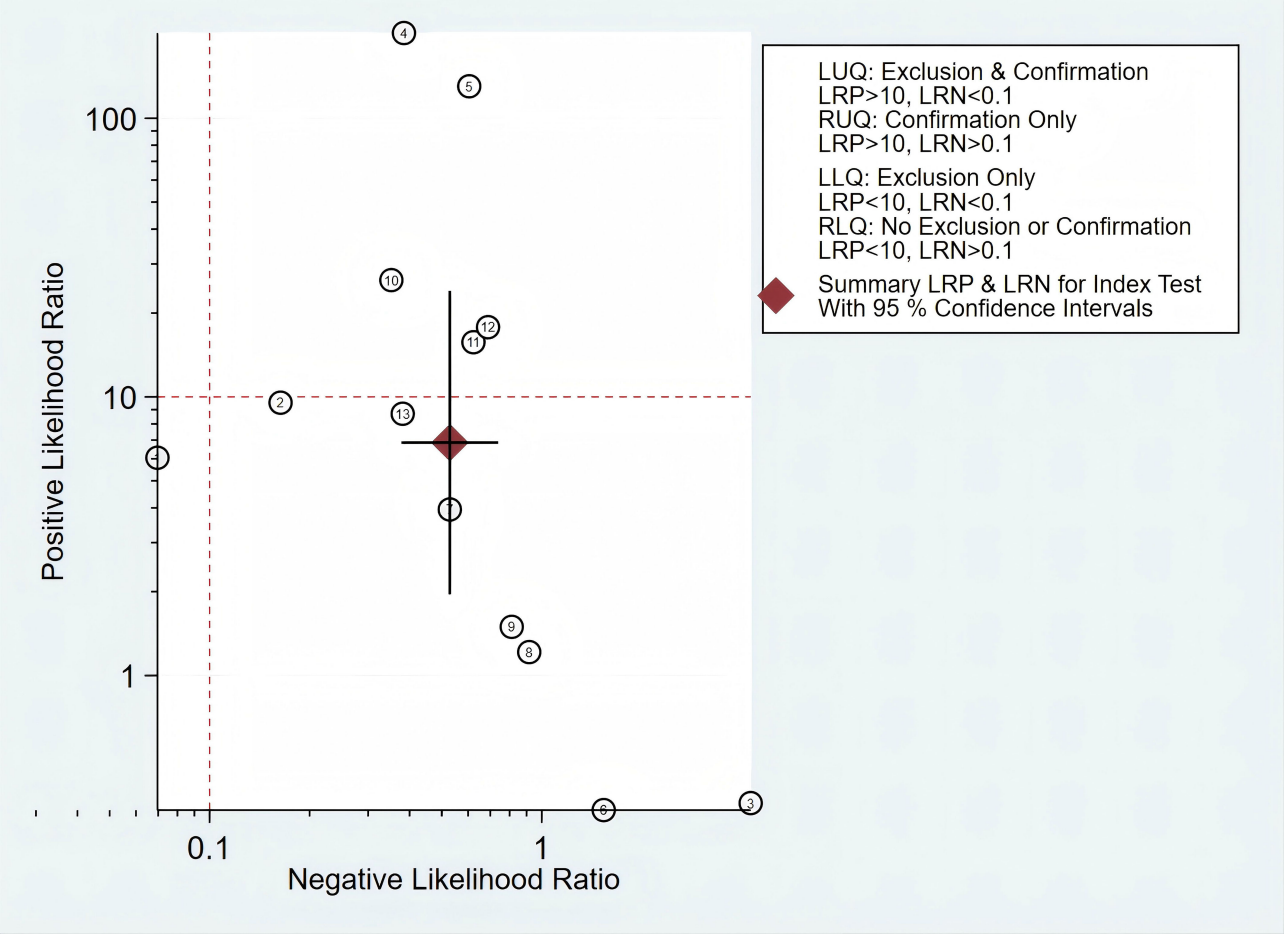
The likelihood ratio scattergram showing the different clinical significances of *mSEPT9* in primary liver cancer.

In addition, we constructed a likelihood ratio scattergram, evaluating the clinical applicability of *mSEPT9* in the diagnosis of PLC (**Figure 7**). The summary likelihood ratios of the *mSEPT9* test are located in the lower right quadrant, indicating that *mSEPT9* did not reach the pathological standard for exclusion and confirmation, thus limiting its clinical utility.

### Sensitivity analyses

Based on the sensitivity analysis, the overall effect estimates showed minimal changes when each study was sequentially excluded, indicating that the meta-analysis results are stable (**Supplementary Figure S2**).

## Discussion

This analysis summarized the diagnostic performance of *mSEPT9* for liver cancer, emphasizing its moderate sensitivity and high specificity. While *mSEPT9* demonstrated strong performance in excluding the disease, it was not sufficient on its own to detect all positive cases within the patient cohort. Therefore, combining *mSEPT9* with other diagnostic methods, such as imaging techniques or additional biomarkers, was essential to improve the detection rate of positive cases. We observed substantial heterogeneity in diagnostic metrics across studies, suggesting potential variability in test application or differences in study populations. The presence of publication bias, as suggested by Deeks’ funnel plot, indicated that smaller studies with potentially overestimated effects might have been more likely to be published. These results underscored the potential utility of *mSEPT9* in liver cancer diagnostics but also emphasized the need for careful consideration of the test’s limitations and the contexts of its use.

DNA methylation is a critical component of the complex epigenetic transcriptional regulation system that is often altered in cancer, leading to hypermethylation in promoter sequences and hypomethylation across genomic sequences (27). *SEPT9* gene methylation has emerged as a significant biomarker in oncology, primarily due to its role in regulating gene expression through epigenetic mechanisms (28). Some studies suggest that *mSEPT9* demonstrates diagnostic potential in various types of cancer. A meta-analysis including 19 case-control studies assessed the diagnostic performance of *mSEPT9* in early colorectal cancer (CRC) screening, finding that *mSEPT9* testing has high specificity (92%) and moderate sensitivity (69%) (29). In recent years, researchers have also begun to focus on the diagnostic efficacy of *mSEPT9* in breast cancer patients, finding that it has higher specificity and lower sensitivity, with diagnostic accuracy slightly superior to that of carcinoembryonic antigen, cancer antigen 153, and cytokeratin 19 (30). In addition, Powrózek et al. evaluated plasma *mSEPT9* in 70 cancer patients and 100 healthy individuals, finding a sensitivity of 44.3%, specificity of 92.3%, and a positive predictive value of 91.2%. They observed higher methylation rates in non-small cell lung cancer and squamous cell carcinoma compared to small cell lung cancer (31). *mSEPT9* has emerged as a promising biomarker for cancer diagnosis, and is associated with clinical features such as TNM staging, tumor size, and overall survival time (32).

However, despite its potential, the diagnostic performance of *mSEPT9* for liver cancer remains unclear. *SEPT9* is typically hypermethylated in liver cancer, which suppresses its expression. This gene plays a critical role in liver functions, such as the development of lipid droplets associated with cirrhosis and non-alcoholic steatohepatitis (NASH), as well as in cellular processes like apoptosis and hepatic stellate cell activation, which are crucial for liver fibrogenesis and carcinogenesiss (19). Liquid biopsy analysis, including circulating cfDNA analysis, offers the opportunity to detect DNA methylation markers such as *SEPT9* in a non-invasive manner for patients with liver disease (33). Given that previous studies have highlighted unsatisfactory sensitivity, advancements in testing methodologies have led to the development of more sensitive assays, such as co-amplification at lower denaturation temperature-polymerase chain reaction (COLD-PCR), which improves detection efficiency without the need for bisulfite treatment of DNA and can detect extremely low levels of methylated DNA (34). Furthermore, the combination of *mSEPT9* and other biomarkers (such as AFP) has been emphasized in recent research, not only to increase sensitivity for malignant tumor diagnosis but also to predict overall survival, microvascular invasion, and tumor proliferation (26). Importantly, a model to predict outcomes in HCC patients treated with molecular targeted agents, combining *mSEPT9* with clinical parameters, demonstrated good predictive ability (35, 36). Previous studies have shown that *mSEPT9*, along with a panel including other epigenetic markers such as RASSF1A and other methylation gene profiles, was able to improve sensitivity and specificity (37, 38). We also observed a multiplex marker panel, including *SEPT9* and multiple HCC-specific markers, as a training panel that can identify HCC-specific methylation patterns with high sensitivity using next-generation sequencing (24). Besides, the combination of multi-omics approaches incorporating genomics, epigenomics, transcriptomics, proteomics, and metabolomics has been highlighted as essential for advancing liver cancer screening. These comprehensive strategies may remedy the limitations of single-marker tests for HCC by considering the intricate molecular landscape of the disease, enabling a more detailed and precise diagnosis (39–41). Of note, some of the trials included in our study suggested that *mSEPT9* performed better in detecting advanced liver cancer compared to early-stage detection (18, 21, 26). Clearly, these studies indicated the need to develop new techniques and methods for *mSEPT9* detection, especially focusing on enhancing its performance in the early stages of cancer.

There were several limitations that should be noted. First, the studies included in this analysis had bias risks in certain areas, particularly in patient selection, which may have impacted the results. Second, the included studies exhibited variations in interventions, blood volume, and measuring method, which may limit the generalizability of the results. Third, not all studies in the meta-analysis provided the specific cutoffs used for classifying *SEPT9* marker-based outcomes. Such heterogeneity in these measurements might be associated with the varying diagnostic accuracy as reported by these different studies, simply because the different cutoff values led to different sensitivity and specificity outcomes. Fourth, HCC and ICC have distinct pathological and molecular profiles, and their amalgamation might have obscured specific diagnostic insights that could be unique to each. Fifth, although *SEPT9* methylation was a biomarker for liver cancer, it may have also been positive in other gastrointestinal cancers such as colorectal, gastric, and esophageal cancers. Studies often selected control groups without these cancers to avoid interference, which limited its real-world applicability. Finally, Deeks’ funnel plot suggested publication bias, indicating that smaller studies with potentially exaggerated effects were more likely to be published, which could have led to an overestimation of mSEPT9’s sensitivity; future larger, multicenter trials should prioritize publishing negative or inconclusive results to reduce this bias.

## Conclusions

This meta-analysis showed that *mSEPT9* testing has high specificity and moderate sensitivity for detecting primary liver cancer, with significant variability in performance metrics across different subgroups. This variability limited its use as an independent screening tool in clinical settings. Future research is urgently needed to enhance the sensitivity of *mSEPT9* and to explore its integration with other diagnostic tools, aiming to improve its cancer screening efficacy.

## Data Availability

The datasets presented in this study can be found in online repositories. The names of the repository/repositories and accession number(s) can be found in the article/**Supplementary Material**.

## Supplementary material

Supplementary Figure 1 Quality assessment result of the included studies.

Supplementary Figure 2 Results of sensitivity analysis.
