# Supplementary figures and images for: Diagnostic accuracy of methylated SEPT9 for primary liver cancer: a systematic review and meta-analysis
Source: Front Endocrinol (Lausanne). 2025 Feb 13;16:1434174. doi: 10.3389/fendo.2025.1434174 (PMC11864958; doi:10.3389/fendo.2025.1434174)

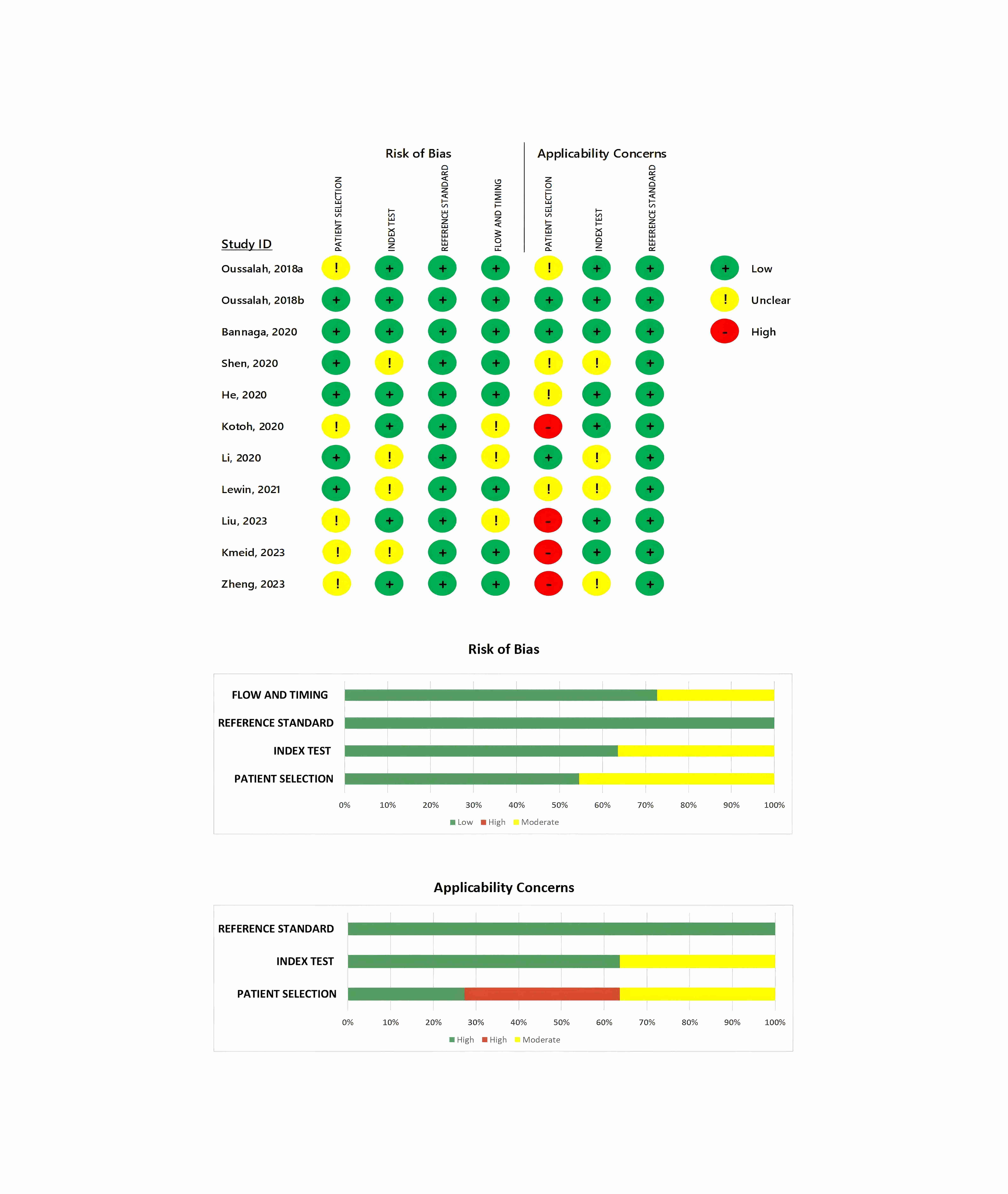

Supplement: Supplementary Figure 1 — Quality assessment result of the included studies. [file Image1.jpeg]

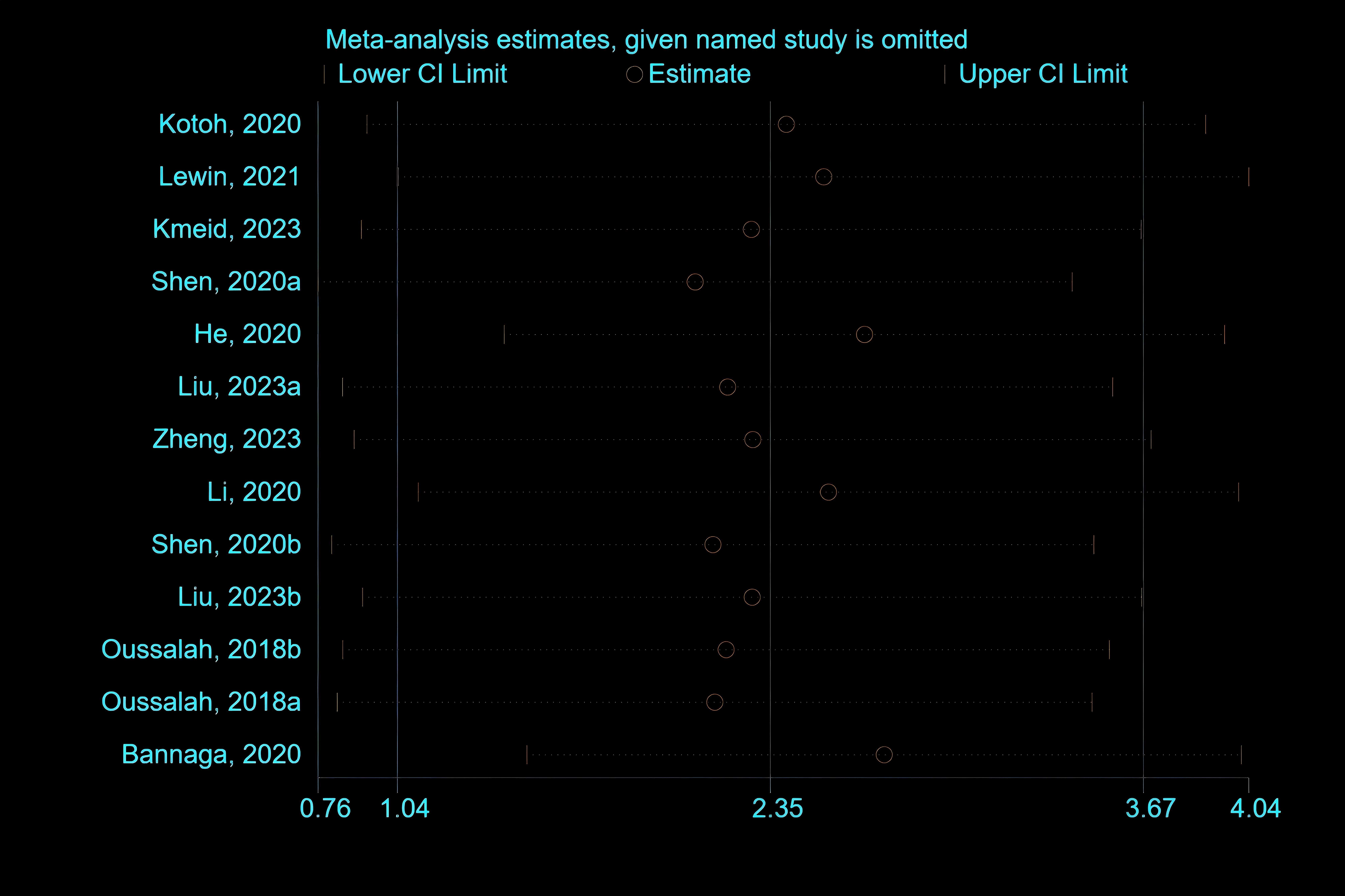

Supplement: Supplementary Figure 2 — Results of sensitivity analysis. [file Image2.jpeg]
