# Supplementary material for: Diagnostic accuracy of methylated SEPT9 for primary liver cancer: a systematic review and meta-analysis
Source: Front Endocrinol (Lausanne). 2025 Feb 13;16:1434174. doi: 10.3389/fendo.2025.1434174 (PMC11864958; doi:10.3389/fendo.2025.1434174)
Supplement: Supplementary file 3 [file Table1.docx]

**Table S1. Literature Search Strategy**

**PubMed**:

- - (("methylated SEPT9" or "SEPT9 methylation" or "SEPT9") AND ("liver neoplasms" OR "liver cancer" OR "hepatocellular carcinoma"))

**Cochrane Library**:

- - "methylated SEPT9" OR "SEPT9 methylation" AND "liver neoplasms" OR "liver cancer" OR "hepatocellular carcinoma" AND "diagnostic accuracy" OR "sensitivity and specificity" in Title Abstract Keyword

**Embase**:

- - ('methylated SEPT9' OR 'SEPT9 methylation') AND ('liver neoplasms'/exp OR 'liver cancer'/exp OR 'hepatocellular carcinoma'/exp) in all field

**CNKI** (China National Knowledge Infrastructure):

- - (("methylated SEPT9") OR ("SEPT9 methylation" OR "SEPT9")) AND (("liver neoplasms") OR ("liver cancer") OR ("hepatocellular carcinoma"))
