# Supplementary material for: Diagnostic accuracy of methylated SEPT9 for primary liver cancer: a systematic review and meta-analysis
Source: Front Endocrinol (Lausanne). 2025 Feb 13;16:1434174. doi: 10.3389/fendo.2025.1434174 (PMC11864958; doi:10.3389/fendo.2025.1434174)
Supplement: Supplementary file 4 [file Table2.docx]

**Table S2. Quality Assessment of each study**

|  | **Risk of Bias** | | | | **Applicability Concerns** | | |
| --- | --- | --- | --- | --- | --- | --- | --- |
| **Study ID** | **PATIENT SELECTION** | **INDEX TEST** | **REFERENCE STANDARD** | **FLOW AND TIMING** | **PATIENT SELECTION** | **INDEX TEST** | **REFERENCE STANDARD** |
| Oussalah, 2018a | Unclear | Low | Low | Low | Unclear | Low | Low |
| Oussalah, 2018b | Low | Low | Low | Low | Low | Low | Low |
| Bannaga, 2020 | Low | Low | Low | Low | Low | Low | Low |
| Shen, 2020 | Low | Unclear | Low | Low | Unclear | Unclear | Low |
| He, 2020 | Low | Low | Low | Low | Unclear | Low | Low |
| Kotoh, 2020 | Unclear | Low | Low | Unclear | High | Low | Low |
| Li, 2020 | Low | Unclear | Low | Unclear | Low | Unclear | Low |
| Lewin, 2021 | Low | Unclear | Low | Low | Unclear | Unclear | Low |
| Liu, 2023 | Unclear | Low | Low | Unclear | High | Low | Low |
| Kmeid, 2023 | Unclear | Unclear | Low | Low | High | Low | Low |
| Zheng, 2023 | Unclear | Low | Low | Low | High | Unclear | Low |
